# Nitric Acid in Hooping Cough and Asthma

**Published:** 1852-07

**Authors:** F. C. T. Arnoldi

**Affiliations:** Lecturer on Midwifery and Diseases of Women and Children, St. Lawrence School of Medicine, Montreal, &c., &c.


From the Canada Medical Journal.

*On Nitric Acid in Hooping Cough and Asthma.* By F. C. T. Arnoldi, M. D

Lecturer on Midwifery and Diseases of Women and Children, St. Lawrence School of Medicine, Montreal, &c., &c.

The few following remarks I take the liberty of communicating to the profession, through the pages of this excellent Journal, feeling perfectly confident they will be read with pleasure, inasmuch as they are somewhat novel as regards the alleviation of hitherto supposed intractable diseases, viz.: hooping cough and asthma. The modus operandi of the remedy, I will not at present attempt to explain, but from the results of my own practice and that of my medical confreres who have watched it and adopted it, I confidently recommend its application to all such as meet with similar cases. In hooping cough, at whatever age, whether it be a child at the breast, or a full grown adult, I administer nitric acid in solution, as strong as lemon juice, sweetened ad libitum. I have given to a child of two years of age, as much as one drachm and a half of concentrated nitric acid, in the above manner per diem, and I have never known the disease to resist its use beyond three weeks. In one instance, that of a child at the breast, only seven months old, the disease disappeared within eight days. In another instance of a young lady fifteen years of age the paroxysms were subdued within the first twenty-four hours, and the disease disappeared within ten days. Again, in the cases of two boys about ten years of age living at a great distance from one another, who had had the cough for several weeks, and to such a violent degree, that both of them had the circumference of their eyes ecchymosed as though they had been pummeled in pugilistic combats, the acid acted positively like a miracle. A medical confrere of mine had four of his children severely affected with the same disease in the middle of winter, and although they had to be kept in-doors owing to the inclemency of the weather, they were nevertheless all perfectly cured within three weeks. I might go on to cite a hundred similar instances, but these, I am satisfied, will prove sufficient to induce the profession to adopt this treatment. As regards asthma, the use of nitric acid has proved not only in my own practice, but in that of others who have adopted it, truly marvellous, and I trust that the profession will remain satisfied by my quoting two special cases. One is that of an elderly person, who had been for five years a frequent inmate of the Montreal General Hospital, a thorough victim to this disease. He generally remained under treatment the winter, and used to be discharged when the disease seemed to have exhausted itself. This patient, about eighteen months ago, was again admitted into the Hospital, under the care of my friend Dr. David, who, observing the obstinacy of the paroxysms, resolved on trying the use of nitric acid; the result was that the first night was passed tolerably; the second night he slept well; the day after the third night he reported himself perfectly convalescent; and on the fifth day he was discharged at his own request, since which he has never been heard of. The other case is that of a stout plethoric servant girl, about thirty-five years of age, who applied to me in the early part of December last. She was then laboring under very severe asthmatic distress, and told me that she had been a martyr to repeated attacks, equally severe for four or five years past; that she had consulted many medical men, but could never obtain any relief, until, as she said, the disease had spent itself. I gave her a prescription containing half an ounce of concentrated nitric acid, and I have never seen her since; but during the New Year holidays, happening to call at the house where she served, I made inquiry about her, when I was told, much to my merriment, that the reason why she never came back to see me was that she thought I had bewitched her. She had often taken medicines which gave her no relief, but that the first night after taking the acid, she slept perfectly sound, and had not, up to that time, had any return of the symptoms. Now, these are obstinate facts, and I trust that this familiar method of communicating them will not diminish their value, nor need any of the profession to be too skeptical to follow the treatment.

